# Hyperactivation of mTOR/eIF4E Signaling Pathway Promotes the Production of Tryptophan‐To‐Phenylalanine Substitutants in EBV‐Positive Gastric Cancer

**DOI:** 10.1002/advs.202402284

**Published:** 2024-07-12

**Authors:** Zi‐Qi Zheng, Cheng‐Rui Zhong, Cheng‐Zhi Wei, Xiao‐Jiang Chen, Guo‐Ming Chen, Run‐Cong Nie, Ze‐Wei Chen, Fei‐Yang Zhang, Yuan‐Fang Li, Zhi‐Wei Zhou, Yong‐Ming Chen, Ye‐Lin Liang

**Affiliations:** ^1^ Department of Gastric Surgery State Key Laboratory of Oncology in South China Guangdong Provincial Clinical Research Center for Cancer Sun Yat‐sen University Cancer Center Guangzhou 510060 P. R. China; ^2^ Department of General Surgery Hepatobiliary Pancreatic and Splenic Surgery The Sixth Affiliated Hospital Sun Yat‐sen University Guangzhou 510655 P. R. China; ^3^ Department of Radiology Oncology State Key Laboratory of Oncology in South China Guangdong Provincial Clinical Research Center for Cancer Sun Yat‐sen University Cancer Center Guangzhou 510060 P. R. China

**Keywords:** aberrant mRNA translation, codon reassignment, EBV‐positive gastric cancer, substitutants, tryptophan

## Abstract

Although messenger RNA translation is tightly regulated to preserve protein synthesis and cellular homeostasis, chronic exposure to interferon‐γ (IFN‐γ) in several cancers can lead to tryptophan (Trp) shortage via the indoleamine‐2,3‐dioxygenase (IDO)‐ kynurenine pathway and therefore promotes the production of aberrant peptides by ribosomal frameshifting and tryptophan‐to‐phenylalanine (W>F) codon reassignment events (substitutants) specifically at Trp codons. However, the effect of Trp depletion on the generation of aberrant peptides by ribosomal mistranslation in gastric cancer (GC) is still obscure. Here, it is shows that the abundant infiltrating lymphocytes in EBV‐positive GC continuously secreted IFN‐γ, upregulated IDO1 expression, leading to Trp shortage and the induction of W>F substitutants. Intriguingly, the production of W>F substitutants in EBV‐positive GC is linked to antigen presentation and the activation of the mTOR/eIF4E signaling pathway. Inhibiting either the mTOR/eIF4E pathway or EIF4E expression counteracted the production and antigen presentation of W>F substitutants. Thus, the mTOR/eIF4E pathway exposed the vulnerability of gastric cancer by accelerating the production of aberrant peptides and boosting immune activation through W>F substitutant events. This work proposes that EBV‐positive GC patients with mTOR/eIF4E hyperactivation may benefit from anti‐tumor immunotherapy.

## Graphical abstract

EBV‐positive gastric cancer (GC) exhibits IFN‐γ‐mediated tryptophan exhaustion within a specialized immune microenvironment. Under excessive activation of the mTOR/eIF4E pathway, tryptophan depletion leads to numerous tryptophan‐to‐phenylalanine (W>F) substitution events, which not only impair protein function but also present abnormal peptides on the cell surface, activating T cells. Therefore, EBV‐positive GC patients with hyperactivated mTOR/eIF4E pathways may manifest better responses to immunotherapy.

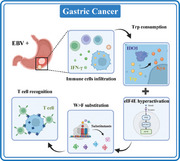

## 1 Introduction

Gastric cancer (GC) is a type of malignant tumor derived from the stomach mucous epithelium, with higher mortality and morbidity rates among all cancers worldwide. Most patients with GC are diagnosed at an advanced stage, with a dismal 5‐year overall survival rate of about 30%.^[^ ^1^ ^]^ In recent years, the immunotherapy represented by immune checkpoint inhibitors (ICI) has brought significant clinical breakthroughs in GC.^[^ ^2^, ^3^ ^]^ However, only a small subset of GC patients is sensitive to, and benefit from, the immunotherapy, and the prognosis of GC patients remains unfavorable.^[^ ^4^ ^]^ Therefore, there is an urgent need to identify the characteristics of the immune microenvironment and uncover the mechanisms of immunotherapy resistance in gastric cancer.

According to the Cancer Genome Atlas (TCGA) network, gastric cancer has been identified into four distinct subtypes by genetic alterations, including microsatellite‐unstable/instability (MSI), genomically stable, chromosomal instability and Epstein‐Barr virus (EBV)‐positive.^[^ ^5^ ^]^ Notably, EBV‐positive GC is characterized by immune inflammation, exhibiting abundant B cell and T cell infiltration. Compared with the EBV‐negative GC, EBV‐positive GC has a better response to immunotherapy and better survival, which may be relevant to its abundant infiltrating lymphocytes and higher PD‐1 expression.^[^ ^6^, ^7^ ^]^ In this regard, gaining a comprehensive understanding of the immunotherapy response using EBV‐positive GC as an entry point is expected to improve the effect of immunotherapy and patient survival.

Extensive tumor inflammation shows hyperactivation of interferon‐γ (IFN‐γ) signaling, leading to elevated indoleamine 2, 3‐dioxygenase 1 (IDO1) expression.^[^ ^8^ ^]^ High IDO1 level causes tryptophan (Trp) consumption via the kynurenine (Kyn) pathway. Both local deprivation of Trp supplement and toxic catabolites hamper the tumor immunity in the microenvironment.^[^ ^9^, ^10^ ^]^ However, cutting‐edge research has demonstrated that cancer cell sustains protein synthesis through ribosomal frameshifting or codon reassignment even with the shortage of Trp.^[^ ^11^, ^12^ ^]^ Ribosomes move backward or forward on the messenger RNA (mRNA) during frameshifting to recover translation in another reading frame,^[^ ^13^ ^]^ which was originally found in some viral genes.^[^ ^14^ ^]^ Interestingly, codon reassignment alters the protein‐coding rules and thus retains in‐frame protein synthesis.^[^ ^15^ ^]^ Both frameshifting and codon reassignment are limited to Trp deficiency in human cells, however, the production rule and regulation mechanism warrant in‐breadth and in‐depth explorations.

Generally, the resources that cells invest to ensure translational accuracy imply that mistranslated proteins are deleterious to cell fitness. Mistranslated proteins may misfold, aggregate, or be involved in the wrong interaction or degradation.^[^ ^16^ ^]^ Since proteins derived from ribosomal frameshifting or codon reassignment belong to cryptic and non‐canonical translation products, they are likely to be rapidly degraded and presented by human leukocyte antigen molecules and promote immune recognition.^[^ ^11^, ^17^, ^18^ ^]^ Currently, abnormal peptides derived by Trp shortage have been identified in various types of malignant solid tumors, including laryngeal squamous cell carcinoma (LSCC), pancreatic ductal adenocarcinoma (PDAC), hepatocellular carcinoma (HCC), head and neck squamous cell carcinoma (HNSCC),^[^ ^19^, ^20^, ^21^, ^22^ ^]^ and were confirmed to be potentially immunogenic,^[^ ^11^, ^12^ ^]^ indicating a ubiquitous phenomenon in human cancers and potential therapeutic strategies across tumors. However, the effect of Trp depletion on the production of abnormal peptides in gastric cancer is still vague.

The present study showed that the EBV‐positive GC has a higher level of infiltrating lymphocytes, including CD4 and CD8+ T cells, and CD20+ B cells, compared with the EBV‐negative GC. The infiltrating lymphocytes continuously secrete IFN‐γ and upregulate IDO1 expression in EBV‐positive GC tissues, which led to a substantial decrease in the Trp level. In addition, Trp deficiency not only caused translation abnormalities in EBV‐positive GC but also induced W>F substitution, while an out‐of‐frame translation event was rare. The existence of abundant W>F substitutants, which also caused T cell activation in EBV‐positive GC, was further verified using a large proteomics dataset from CPTAC‐STAD (PDC000214) and EBV‐positive GC samples. Further analysis revealed that hyperactivation of mTOR‐eIF4E (eukaryotic initiation factor 4E) pathway reinforced the production of W>F peptides and their presentation on the cell surface. These data demonstrated that hyperactivation of mTOR/eIF4E exposed vulnerability by boosting the production of aberrant peptides and immune activation through W>F substitution. We also identified that the EBV‐positive GC with excessive activation of mTOR/eIF4E pathway may have a better therapeutic effect on PD‐1 inhibitors.

## 2 Results

### 2.1 IFN‐γ Induces Trp Consumption by Upregulating IDO1 Expression in EBV‐Positive GC

**Figure 1:**
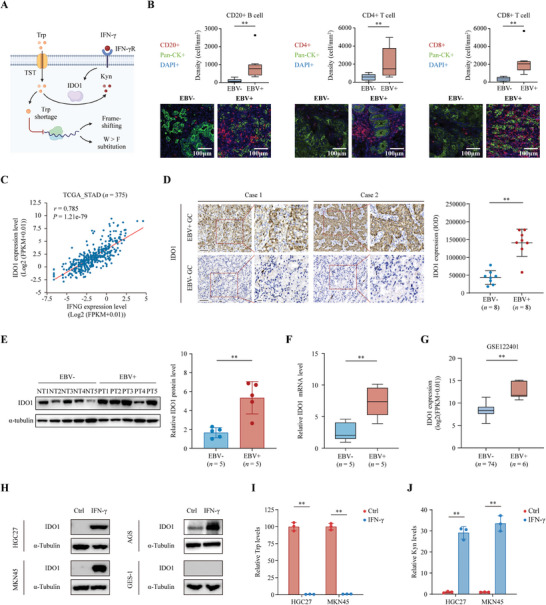
IFN‐γ upregulated IDO1 expression and caused tryptophan exhaustion in EBV‐positive gastric cancer. A) The model diagram showing the effect of IFN‐γ on the generation of ribosomal abnormal peptides. B) The representative images (up panel) and quantification results (bottom panel) of multiplex immunofluorescence staining showing the CD20+ B cell, CD4+ and CD8+ T cell infiltration in 8 pairs of EBV‐negative (NT1‐8) and EBV‐positive (PT1‐8) GC tissues. C) Correlation analysis between mRNA expression of IFNG and IDO1 in TCGA cohort of stomach adenocarcinoma (STAD) patients (*n* = 375). D) Representative images showing IDO1 staining in EBV‐positive GC tissues (*n* = 8, PT1‐8) and paired EBV‐negative GC tissues (*n* = 8, NT1‐8) (left panel). The integrated optical density (IOD) of positive expression cells in 10 cases of GC tissues, and grading of the level of immunoreactivity (right panel). Scale bar = 100 µm. E) The expression level of IDO1 protein was quantified by Western blotting in 5 pairs of EBV‐positive (PT1‐5) and EBV‐negative GC tissues (NT1‐5). F) The expression of IDO1 in other 6 pairs of EBV‐positive and EBV‐negative GC tissues as determined by qPCR. G) Comparison of IDO1 expression level between EBV‐negative GC (*n* = 74) and EBV‐negative GC (*n* = 6) using the gene expression profile data from GSE122401 (*n* = 80). H) Western blotting evaluation of the changes in IDO1 expression in HGC27, MKN45, AGS and GES1 cells in response to IFN‐γ treatment. I, J) The tryptophan (I) and kynurenine (J) levels in HGC27 and MKN45 cells under mock or IFN‐γ treatment were determined using LC‐MS/MS. Data are presented as the mean ± SD. *P < 0.05, **P < 0.01.

IFN‐γ‐mediated upregulation of IDO1 induces Trp consumption, which ultimately induces ribosomal frameshifting and tryptophan‐to‐phenylalanine (W>F) substitution (**Figure** **1**A). Since the EBV‐positive GC has a distinct immune‐microenvironment, with dense infiltration of lymphocytes that secret a large number of cytokines.^[^ ^23^, ^24^ ^]^ We first conducted multiple immunofluorescence (mIF) to assess the tumor‐associated lymphocytes in 8 pairs of EBV‐positive and EBV‐negative GC tissues. As shown in Figure 1B, the density of CD20^+^ B cells, and CD4^+^ and CD8^+^ T cells was apparently higher EBV‐positive GC than EBV‐negative GC. Moreover, analysis of the TCGA gastric cancer database (*n* = 375) showed that expression of IFNG was positively correlated with IDO1 expression in GC (Figure 1C). To assess the expression pattern of IDO1 in EBV‐positive and EBV‐negative GC patients, we collected 8 pairs of EBV‐positive and EBV‐negative GC tissues. Immunohistochemistry (IHC) staining and western blotting analysis revealed that IDO1 was highly expressed in EBV‐positive GC tissues compared with EBV‐negative tissues (Figure 1D,E). Similar results were observed in another 6 pairs of GC tissues using quantitative real‐time PCR (qRT‐PCR) analysis (Figure 1F and *P* < 0.01), and the transcriptome profiling of 74 EBV‐negative GC tissues and 6 EBV‐positive GC tissues from the Gene Expression Omnibus (GEO) dataset (GSE122401) (Figure 1G and *P* < 0.01). Gene set enrichment analysis (GSEA) based on the transcriptome profile of GSE122401 revealed stronger dominance of IFN‐γ, Trp metabolism, and T cell activation in EBV‐positive GC (Figure S1A‒C, Supporting Information). We then examine the effect of IFN‐γ on IDO1 expression in GC cell lines. As anticipated, IFN‐γ treatment led to a massive upregulation of IDO1 expression in HGC27 and MKN45 cells, thereby invoking the exhaustion of Trp and accumulation of its metabolism product, Kyn. This phenomenon was not observed in immortalized gastric epithelial cell line (GES1) (Figure 1H‒J). Collectively, these data revealed that EBV‐positive GC showed stronger inflammatory microenvironment and higher level of IFN‐γ that boosted IDO1 expression and therefore caused Trp consumption.

### 2.2 Trp Depletion Leads to W>F Substitutions in GC Cells

**Figure 2:**
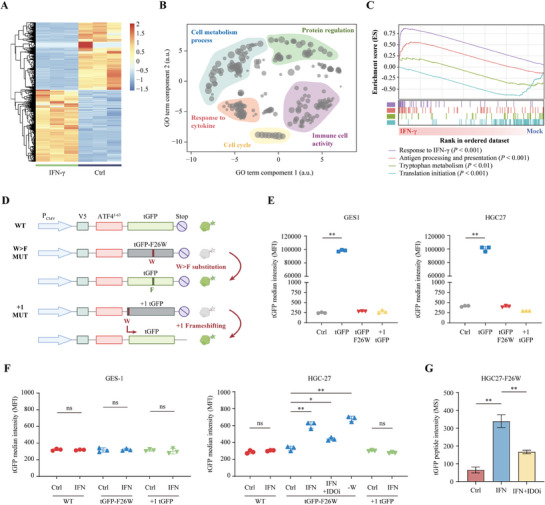
Tryptophan deficiency resulted in tryptophan‐to‐phenylalanine substitution translation in GC cells. A) Cluster heatmap of the differentially expressed genes in IFN‐γ treated MKN45 cells compared to the mock treated MKN45 cells. B) REVIGO^[^ ^48^ ^]^ summary of the different Gene Ontology (GO) terms between mock‐ and IFN‐γ treated MKN45 cells. C) GSEA plots of the enrichment pathway associated with IFN‐γ treatment in MKN45 cells. D) This figure illustrates the constructs utilized to identify frameshifting and substitution translation events in GC cell. The Wild Type (WT) vector, containing a V5‐tag (blue box), was linked to ATF4^1–63^ (red box, featuring tryptophan residue W at the 93^rd^ amino acid from the translation starting site) and positioned upstream of TurboGFP (tGFP, green box, with fewer tryptophan residues). The W>F substitution construct (W>F MUT) was derived from the WT vector, where the phenylalanine residue at position 26 was altered to tryptophan (F26W). The constructs for detecting frameshifting events (+1 MUT) were also transformed from the WT, with one additional base pair upstream the tGFP and leading to an out‐of‐frame tGFP product. E) The tGFP signals in GES1 and HGC27 cells stably expressing WT, W>F MUT, or +1 MUT reporters, respectively, were measured by flow cytometry assay. F, G) GES1 and HGC27 cells stably expressing the WT, W>F MUT, or +1 MUT reporters were mock‐treated or treated with IFN‐γ (250U/mL) for 48 h and subjected for flow cytometry analysis (F) or mass spectrometry analysis (G) to measure the tGFP signaling intensity. Data are presented as the mean ± SD. *P < 0.05, **P < 0.01, ns = not significant.

For a better understanding of the comprehensive effects of IFN‐γ in gastric cancer, we performed whole‐genome RNA‐seq of mock‐ and IFN‐γ treated MKN45 cells followed by differential gene expression (**Figure** **2**A, Table S1, Supporting Information) and gene set enrichment analysis. Gene ontology (GO) of their differently expressed genes revealed enrichment of five distinct of semantically related ontology terms, including cell metabolism process, response to cytokine, cell cycle, immune cell activity, and protein regulation (Figure 2B). GSEA results also showed that IFN‐γ treatment was closely related to Trp metabolism, protein translation, and antigen presentation process (Figure 2C). Since Trp shortage was recently demonstrated to cause ribosomal frameshifting and W>F substitution at sites of Trp codons in various types of tumors,^[^ ^11^, ^25^ ^]^ we therefore explored whether this phenomenon also occurs in GC. We engineered constructs of ATF4^1‐63^‐turbo green fluorescent protein (tGFP, WT), W>F substitution reporters (ATF4^1‐63^‐tGFP‐F26W, W>F MUT), and ribosomal frameshifting reporters (ATF4^1‐63^‐+1 tGFP, +1 MUT) (Figure 2D). Then, flow cytometry was used to measure GFP signals in GC cell lines (HGC27 and MKN45) and GES1. The WT cells showed a high intensity of GFP signals that were not present in the W>F MUR or +1 MUT cells (Figure 2E; Figure S2A, Supporting Information). Then, the cells were treated with mock, IFN‐γ (250 U/mL), IFN‐γ, and an IDO inhibitor (IDOi, 0.1 µM), or cultured in a Trp‐free (‐W) medium. No difference in GFP signal intensity was observed for any groups of GES1 cells (Figure 2F, left panel), indicating that there were no frameshifting or substitution events in GES1 cells under IFN‐γ exposure. Conversely, IFN‐γ or ‐W treatment dramatically increased the GFP intensity in HGC27 or MKN45 cells expressing tGFP‐F26W. Meanwhile, the effect of IFN‐γ was attenuated by IDOi treatment (Figure 2F, right panel, Figure S2B,C, Supporting Information). This difference was also confirmed by mass spectrometry (MS) for GFP protein abundance (Figure 2G, all *P* < 0.01). Taken together, these results demonstrated that IFN‐γ‐mediated Trp deficiency mainly induced W>F substitution in GC cells.

### 2.3 Identifying the Codon Reassignment Events in GC Cell Lines by Trp Shortage

**Figure 3:**
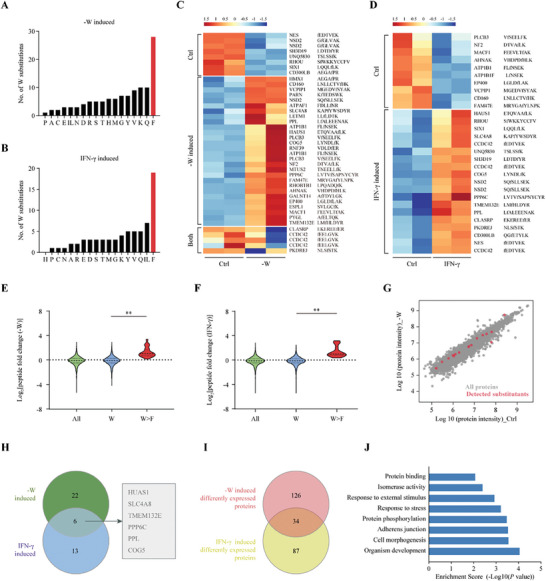
Detection of the endogenous W>F substitutants in GC cell lines under tryptophan shortage. A, B) Bar plots showing the cumulative number of tryptophan substitutants identified by mass spectrometry analysis in MKN45 cells cultured in tryptophan‐free medium (A) or treated with IFN‐γ (250U/mL) for 48 h (B). C, D) Heatmaps displaying of the relative abundance for W>F substitutants in MKN45 cells cultured in control, tryptophan‐free medium (C), or treated with IFN‐γ (250U/mL) for 48 h (D). Data were obtained from two biological replicates. The detected W>F substitutants were divided into three groups, Ctrl (only detected in control cells), ‐W or IFN‐γ induced (only detected in ‐W or IFN‐γ treatment cells), and both (detected in both groups). E, F) The violin plots illustrating the relative peptide abundance between the control and ‐W treatment condition (E), or the control and IFN‐γ treatment condition (F), for all peptides in MKN45 cells proteome. These groups comprised either all the peptides detected in the entire proteome (All), peptides that encompassed the tryptophan codon and included tryptophan (W), or those with W>F substitutions (W>F), respectively. G) Scatter plot showing the relative protein expression levels (log10 protein intensity) from mass spectrometry analysis in control (x‐axis) and ‐W treated (y‐axis) MKN45 cells for all (grey dots) or W>F substitutants (red dots) peptides. H) Venn diagram showing the intersection of W>F substitutants detected in MKN45 cells with ‐W or IFN‐γ treatment. I) Venn plot for the intersection of differently expressed proteins in MKN45 cells under ‐W or IFN‐γ treatment. J) The differently expressed genes in MKN45 cells under ‐W or IFN‐γ treatment were submitted for GO and KEGG enrichment analyses. Data are presented as the mean ± SD. **P < 0.01.

To first explore the endogenous W>F peptides induced by Trp shortage in the whole‐cell proteome, MKN45 cells were exposed to IFN‐γ or cultured in ‐W medium and then MS was performed with duplicate assays. After rigorous screening, it was found that the number of W>F events after IFN‐γ or ‐W treatment was significantly higher than that of other codon reassignments (**Figure** **3**A,B). Specifically, we detected abnormal W>F peptides derived from 26 genes in MKN45 cells under a ‐W condition (Figure 3C, Table S2, Supporting Information), and W>F peptides from 18 genes in MKN45 cells after IFN‐γ treatment (Figure 3D, Table S3, Supporting Information). Moreover, a high abundance of W>F peptides was observed in the context of global downregulation of protein synthesis during IFN‐γ and ‐W treatment, which partially accounted for the reduced mRNA translation in amino acid‐deficient conditions (Figure 3E,F). Further analysis showed no significant change in the expression levels of these genes after ‐W treatment, suggesting that the occurrence of W>F substitution was not associated with the protein expression level (Figure 3G). Particularly, 6 W>F substitutants were detected in both IFN‐γ‐ and ‐W‐treated MKN45 cells, including HAUS1, SLC4A8, TMEM132E, PPP6C, PPL, and COG5 (Figure 3H). These peptides belonged to protein classes with different functions (e.g., cell survival and division, ions transport, cell development, and vesicular trafficking). Further analysis showed that the WT residues of most proteins were very conservative, and the W>F mutation may be deleterious to the functions of these proteins. We further analyzed the protein expression profiles by quantitative MS and identified 34 differently expressed proteins after Trp depletion and IFN‐γ treatment (Figure 3I, Tables S4,S5, Supporting Information). These proteins were primarily involved in protein phosphorylation, stress and stimulus response, and isomerase activity (Figure 3J). Overall, these results revealed a novel translation mechanism of alternative mRNA decoding under Trp shortage in GC.

### 2.4 EBV‐Positive GC Contained Abundant W>F Substitutants

**Figure 4:**
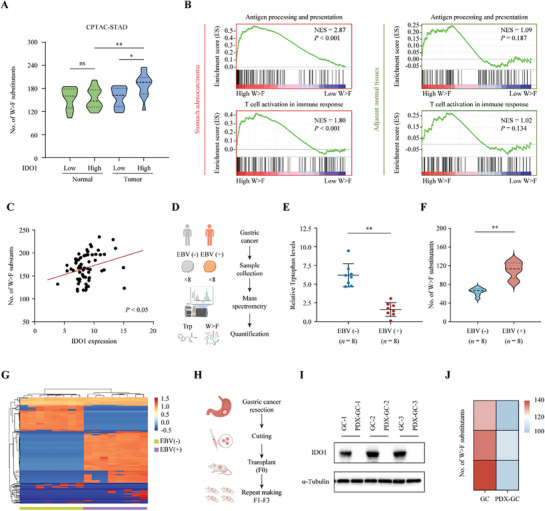
Identification of the W>F substitutants in EBV‐positive GC tissues. A) Violin plots illustrating the number of W>F substitution events in the IDO1‐low and IDO‐high STAD tissues and normal adjacent samples from CPTAC database. B) Samples from the CPTAC‐STAD dataset were classified into high‐ or low‐W>F based on the number of W>F substitution events. GSEA was performed to explore the potential biological functions in the high‐ or low‐W>F groups in both STAD tissues (left panel) and normal adjacent samples (right panel). C) The correlation between IDO1 expression level and number of W>F substitutants was assessed using Pearson correlation analysis. Data was obtained from the CPTAC‐STAD dataset (PDC000214). D‒G) Fresh frozen tissues from 8 pairs of EBV‐positive (PT1‐8) and EBV‐negative GC (NT1‐8) were collected and sent for mass petrography analysis for Trp quantification and W>F substitution analysis. Schematic representation of the workflow (D). Relative Trp level in GC samples was displayed as scatterplot with mean± SD (E). The W>F substitutants abundance in GC samples was visualized as violin plot (F) and heatmap (G). H‒J) PDX was established using 3 cases of fresh EBV‐positive GC tissues after surgical resection. The fresh tissues (labelled as GC1‐3) and the successful engraftments (F3, labelled as PDX‐GC1‐3) were sent for further analysis. The Schematic representation of PDX experimental design (H). Western blot analysis of IDO1 expression was performed in donor and PDX tissues (I). Heatmap showing the W>F substitutants abundance in donor and PDX tissues (J). Data are presented as the mean ± SD. *P < 0.05, **P < 0.01, ns = not significant.

To validate these findings in clinic, we first performed whole‐cell proteome analysis based on the Clinical Proteomic Tumor Analysis Consortium (CPTAC) dataset (https://proteomic.datacommons.cancer.gov/pdc/). The proteogenomics of GC (CPTAC‐STAD, PDC000214) was examined, which contains GCs and adjacent normal tissues from 65 patients (5 cases of EBV‐positive GCs and 60 cases of EBV‐negative GCs).^[^ ^26^ ^]^ Then, GC and normal tissues were grouped according to the IDO1 expression (IDO1 high (top 20%, *n* = 13) and IDO1 low (bottom 20%, *n* = 13)), respectively. As shown in **Figure** **4**A, no substantial difference in the abundance of W>F peptides was detected between the two groups from adjacent normal tissues, while the abundance in the IDO1 high group was higher than that of the IDO low group from GC tissues (Table S6, Supporting Information). We further divided these samples into two groups (high W>F (top 20%, *n* = 13) and low W>F (bottom 20%, *n* = 13)) according to the W>F peptide abundance. GSEA revealed that high W>F peptide abundance was closely associated with the immune response, antigen presentation, T cell activation, NK cell cytotoxicity, cytokine interaction, NK‐κB signal, and mTOR signal pathways. However, no significant difference was found in these pathways from normal tissues (Figure 4B; Figure S3A, Supporting Information). We further conducted a comprehensive analysis of immune cell infiltration abundances in GC tissues using an online tool Tumor Immune Estimation Resource (TIMER, https://timer.cistrome.org/). Compared with the low W>F group, GC tissues with a higher W>F substitution level had greater infiltration of CD8^+^ T cells and cytotoxic T lymphocytes (CTL) (Figure S3B, Supporting Information), indicating that the abundance of W>F peptides was positively associated with high levels of tumor infiltration by activated T‐cells. Moreover, correlation analysis revealed significantly positive associations between IDO1 expression and the number of W>F substitutants (Figure 4C). Taken together, we initially confirmed the existence of W>F substitutants in GC tissues based on the public database.

Subsequently, we collected 8 paired of fresh frozen EBV‐positive and EBV‐negative GC tissues, and conducted MS to quantify the Trp level and search for the endogenous W>F substitutants (Figure 4D). As expected, the Trp level in EBV‐positive GCs was significantly lower compared to the EBV‐negative GCs (Figure 4E). Although a larger number of W>F substitutants were detected in all GC tissues, the abundance of W>F substitutants was much higher in the EBV‐positive GCs (Figure 4F‒G, *P* < 0.01, Table S7, Supporting Information). To support the association between tumor infiltrating lymphocytes and the generation of W>F substitutants, we established 3 cases of patients‐derived EBV‐positive gastric cancer xenografts (PDX) in immunodeficient mice (Figure 4H). Totally, 3 pairs of donors (GC) and PDX (PDX‐GC, F3) tumors were analyzed. Unlike gastric cancers in their natural immune microenvironment, PDX samples did not exhibit elevated IDO1 expression (Figure 4I) or enrichment of W>F substitutants (Figure 4J), illustrating that the emergence of W>F substitutants in gastric cancer did not due to the EB virus, but to the host tumor microenvironment and compatible immune response.

### 2.5 The mTOR/eIF4E Pathway was Engaged with the Generation of W>F Substitutants in EBV‐Positive GC

**Figure 5:**
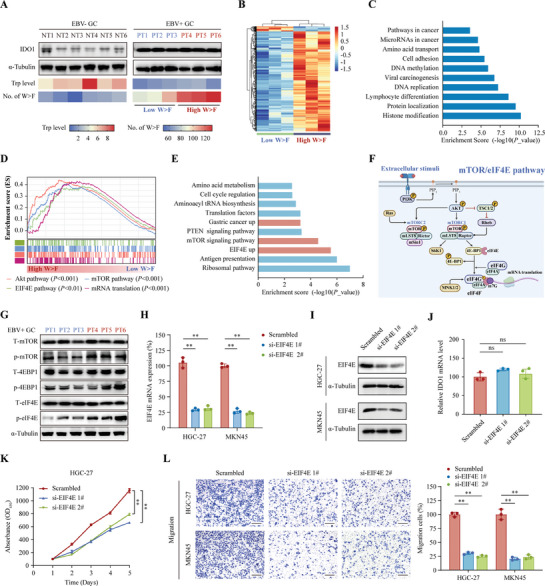
The mTOR/eIF4E pathway was an important turner for the generation of W>F substitutants in EBV‐positive GC. A) 6 cases of EBV‐positive GC tissues with highest (*n* = 3, PT1‐3) and lowest (*n* = 3, PT4‐6) abundance of W>F substitutants were chosen for further analysis. 6 cases of paired EBV‐negative GC tissues served as controls (NT1‐6). IDO1 expression, Trp level, and number of W>F substitutants were shown, respectively. B‒D) The differently expressed genes between High and Low W>F GC tissues were screened from RNA sequencing. Heatmap showing the hierarchical clustering of significant differential genes (|Fold change| > 2, *P* < 0.05) (B). Gene Ontology (GO) analysis of the biological functions and pathways related to the generation of W>F substitutants (C). GSEA result based on the expression profile demonstrating the enrichment of Akt‐mTOR‐eIF4E pathway in the High W>F group (D). E) GO and KEGG functional analysis for the differently expressed genes between High‐ and Low‐ W>F groups based on CPTAC‐STAD (PDC000214) and GEO (GSE122401) datasets. F) Schematic representation of the mTOR/eIF4E pathway illustrates that eIF4E is located downstream of mTOR. G) Western blot analysis showing the level of total mTOR (T‐mTOR), phospho‐mTOR (p‐mTOR), total 4EBP1 (T‐4EBP1), phosphor‐4EBP1 (p‐4EBP1), total eIF4E (T‐eIF4E), and phospho‐eIF4E (p‐eIF4E) in the high W>F and low W>F group of EBV‐positive GC tissues. H, I) HGC27 and MKN45 cells were transfected with siRNAs targeting EIF4E (si‐EIF4E 1# and 2#), and the knockdown efficiency was validated using qPCR (H) and Western blotting analysis (I). J) The mRNA expression level of IDO1 in HGC27 cells was compared between control siRNA and EIF4E siRNAs groups. K) CCK‐8 assay was conducted to evaluate the proliferation ability of HGC27 cells transfected with control siRNA or EIF4E siRNAs. L) After knockdown of EIF4E with two individual siRNAs, the migration and invasion ability of HGC27 cells was detected by Transwell assay. The representative images (left panel) and the counts migratory/invasive cells (right panel) in the Transwell assay. Scale bar = 200 µm. Data are presented as the mean ± SD. **P < 0.01, ns = not significant.

From the above 8 cases of EBV‐positive GC tissues, we observed that although there was no significant difference in the Trp level, the abundance of W>F substitutants was still diverse, suggesting there may be alternative mechanisms regulating the generation of W>F substitutants. To further explore this possibility, we analyzed the above 6 cases of GC tissues with the highest and lowest W>F abundance, respectively (**Figure** **5**A). In RNA sequencing, 1008 significantly differently expressed genes (DEGs) were obtained (|Fold change| > 2, *P* < 0.05), including 967 upregulated genes and 41 downregulated genes (Figure 5B, Table S8, Supporting Information). Gene enrichment analysis revealed that the DEGs mainly involved in DNA replication, viral carcinogenesis, microRNA in cancer, and Pathway in cancer (Figure 5C), indicating that the generation of W>F substitutants may be implicated in cancer cell proliferation. We then conducted GSEA to elucidate the cancer‐related pathways associated with the W>F substitutions. As shown in Figure 5D, GSEA plot depicting Akt‐mTOR‐eIF4E and mRNA translation pathways between high and low W>F substitutants groups. These results were further confirmed by KEGG enrichment analysis based on CPTAC‐STAD dataset (Figure 5E, Table S9, Supporting Information). The mTOR pathway servers as a core regulator of diverse biological functions and signaling pathways in mammal cells, in which eIF4E is one of the key downstream of mTOR to promote protein synthesis (Figure 5F).^[^ ^27^ ^]^ Interestingly, eIF4E is a limiting factor in translation initiation and the overexpression or hyperactivation eIF4E was reported to be associated with malignant transformation in numerous cancer types.^[^ ^28^, ^29^ ^]^ Western blot analysis showed that the phosphorylation level of mTOR (p‐mTOR), 4EBP1 (p‐4EBP1) and eIF4E (p‐eIF4E) was remarkably higher in the high W>F group (T4‐6) than the low W>F group (T1‐3) (Figure 5G). Based on these evidences, we propose a hypothesis that hyperactivation of mTOR‐eIF4E pathway increased the generation of W>F substitutants by accelerating the protein synthesis in EBV‐positive GC.

Given the role of EIF4E in gastric cancer has not been fully elucidated, we first sought to explore whether EIF4E had tumor‐promoting effects in GC. Small interfering RNA (siRNA) technology was employed to silence EIF4E expression in HGC‐27 and MKN45 cells. The knockdown efficiency of each siRNA was determined by qRT‐PCR (Figure 5H) and Western blot analysis (Figure 5I). Notably, EIF4E knockdown had no impact on the IDO1 expression (Figure 5J). Then, colony formation assay, Cell Counting Kit‐8 (CCK‐8) analysis and Transwell assay were used to assess changes in the proliferation, migration, and invasion abilities of HGC‐27 and MKN‐45 cells following EIF4E knockdown. As anticipated, cells with silenced EIF4E exhibited a significantly slower proliferation rate (Figure 5K; Figure S4A,B, Supporting Information). Transwell assays revealed that EIF4E knockdown significantly inhibited the migration and invasion of HGC27 and MKN45 cells (Figure 5L; Figure S4C (Supporting Information), all *P* < 0.01). These results validated the oncofetal role of EIF4E in gastric cancer.

### 2.6 Inhibiting of mTOR/eIF4E Pathway Alleviated the Generation of W>F Substitutants

**Figure 6:**
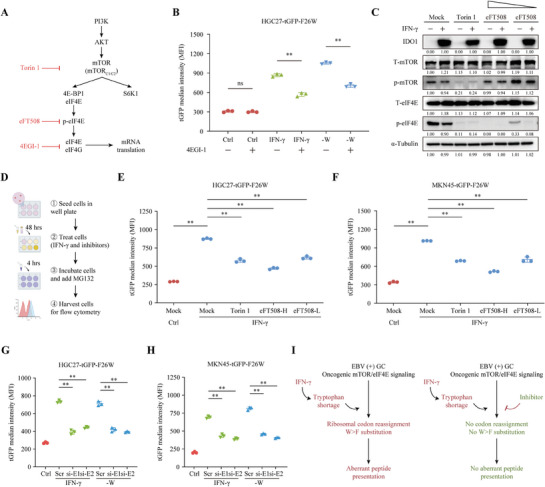
The inhibition of the mTOR/eIF4E pathway attenuate the generation of W>F substitutants in GC. A) A diagram illustrating the site of action of each inhibitor. B) HGC27 cells stably expressing tGFP‐F26W were culture in the normal, IFN‐γ or ‐W medium and treated with 4EGI‐1 (25 µM) for 24 h. Flow cytometry analysis for the tGFP signal intensity in each group. C) Western blot results showing the level of IDO, T‐mTOR, p‐mTOR, T‐eIF4E, and p‐eIF4E in HGC27‐tGFP‐F26W cells treated with IFN‐γ (or control) and various inhibitors as indicated. The eFT508 treatment was performed at two different concentrations (2.5 nM and 10 nM). D‒F) HGC27 and MKN45 cells with tGFP‐F26W stable expression were cultured in medium with Ctrl or IFN‐γ, and treated with Torin 1 or eFT508 (2.5 nM for low concentration and 5 nM for high concentration). Then, MG132 (10 µM) was added to inhibit proteasome and cells were sent for clow cytometry analysis. Schematic flow chart of the experimental design (D). Flow cytometry results for the signal intensity of tGFP in HGC27 (E) and MKN45 (F) cells. G, H) HGC27 (G) and MKN45 (H) cells stably expressing tGFP‐F26W were transfected with control siRNA or EIF4E siRNAs and cultured in control, IFN‐γ or tryptophan‐depletion (‐W) medium. Flow cytometry results showing the signal intensity of tGFP. I) A scheme of the influence of IFN‐γ‐mediated Trp consumption in the generation of W>F substitutants in EBV‐positive GC with mTOR/eIF4E pathway hyperactivation (left panel). Inhibiting mTOR/eIF4E pathway with inhibitors were demonstrated to alleviate the generation and presentation of W>F peptides. Data are presented as the mean ± SD. **P < 0.01, ns = not significant.

To further verify the association between mTOR/eIF4E pathway and the generation of W>F substitutants, we employed various inhibitors to inhibit the mTOR‐eIF4E pathway, including Torin 1 (200 nM) to inhibit mTOR,^[^ ^30^ ^]^ eFT508 (5–10 nM) to inhibit phosphorylation of eIF4E,^[^ ^31^ ^]^ and 4EGI‐1 (25 µM) to inhibit eIF4E/eIF4G interaction and the translation initiation functions of eIF4E^[^ ^32^ ^]^ (**Figure** **6**A). First, HGC27‐tGFP‐F26W cells were cultured in the normal, IFN‐γ or ‐W medium, and exposed to mock or 4EGI‐1. Then, flow cytometry was conducted to analyze the changes in tGFP signal intensity under 4EGI‐1 treatment. As expected, compared with the mock treatment, the inhibitor of eIF4E/eIF4G interaction remarkably decreased the tGFP signal in HGC27 cells (Figure 6B). Furthermore, we conducted Western blot to verify the efficiency of Torin 1 and eFT508 in inhibiting the mTOR/eIF4E pathway. As shown in Figure 6C, treatment of the mTOR inhibitor (Torin 1) caused significant inhibition of mTOR and eIF4E phosphorylation. Moreover, the eIF4E phosphorylation level decreased gradually with increased concentration of the eIF4E inhibitor (eFT508). The tGFP signals in HGC27 and MKN45 cells expressing tGFP‐F26W were monitored by flow cytometry analysis (Figure 6D). As expected, cells with IFN‐γ exposure displayed much stronger tGFP signals, which were impaired by Torin 1 or eFT508 treatment with dose dependence (Figure 6E, F). We then investigated the effects of mTORC1 inhibitor (Rapamycin), mTORC2 inhibitor (JR‐AB2‐011), and mTORC1/C2 inhibitor (Torin 1) on the generation of W>F substitution. The results showed that using an mTORC2 inhibitor partially weakened the production of W>F substitutants under IFN‐γ treatment, whereas the mTORC1 inhibitor significantly reduced the production of W>F substitutants (Figure S5A,B, Supporting Information), suggesting that the mTORC1 was the primary pathway promoting the production of W>F substitutants. Subsequently, we silenced EIF4E expression, a key regulator of mTOR/eIF4E pathway, in HGC27‐ and MKN45‐tGFP‐F26W cells. Flow cytometry analysis showed that cell treated with IFN‐γ or cultured in medium without Trp had stronger tGFP signals than the control group, which could be weaken by EIF4E knockdown (Figure 6G,H). Taken together, these results proclaimed a strong correlation between the hyperactivation of mTOR/eIF4E pathway and the production of W>F substitutants in EBV‐positive GC (Figure 6I).

### 2.7 Aberrant W>F Peptides can be Presented on the Cell Surface and Caused T‐Cell Activation in EBV‐Positive GC

**Figure 7:**
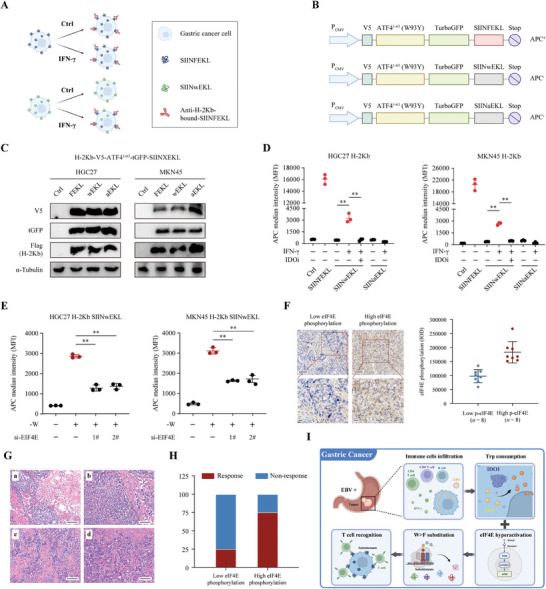
The W>F substitutants could be presented on the cell surface and activate anti‐GC immunity. A) Schematic diagram showing the effect of IFN‐γ on the OVA peptide SIINFEKL model. IFN‐γ treatment increases the presentation and recognition of SIINFEKL or SIINwEKL peptides, which can be detected by anti‐H‐2Kb‐bound antibodies. B) Schematic diagram showing the used to evaluate the production, presentation and recognition of SIINFEKL, SIINwEKL, and SIINaEKL peptides, which were placed downstream of the V5‐ATF4^1–63^(W93Y)‐tGFP. C) HGC27 and MKN45 cells stably expressing flag‐tag H‐2Kb were transfected with empty (Ctrl), tGFP‐SIINFEKL, tGFP‐SIINwEKL, or tGFP‐SIINaEKL vectors. Western blotting verification of similar expression level of the reporters and flag‐tag H‐2Kb using anti‐V5, anti‐tGFP, anti‐Flag, and anti‐α‐Tubulin. D) Flow cytometry analysis of the APC median fluorescence intensity (MFI) of H‐2Kb‐bound SIINFEKL peptides in HGC27 and MKN45 cells stably expressing H‐2Kb and the SIINFEKL reporters. The culture conditions and drug treatments were are shown in the figures. E) HGC27 and MKN45 cells with stable expression of H‐2Kb and SIINwEKL were transfected with control siRNA or EIF4E siRNAs and cultured in the complete or tryptophan‐depletion medium. Flow cytometry analysis was performed to measure the APC median fluorescence intensity (MFI) of H‐2Kb‐bound SIINFEKL peptides. F) The relationship of eIF4E phosphorylation level and immunotherapy response was assessed by IHC staining in an EBV‐positive GC cohort (*n* = 16). The level of p‐eIF4E was measured using the integrated optical density (IOD) system. Patients were categorized into Low p‐eIF4E (*n* = 8) and High p‐eIF4E (*n* = 8) group. Scale bar = 100 µm. G) 16 cases of EBV‐positive GC patients received treatment combining chemotherapy and immunotherapy before surgical resection, and the immunotherapeutic response was evaluated using the tumor regression grade (TRG) system in accordance with the NCCN guideline. TRG 0  =  complete response, TRG 1  =  near complete response, TRG 2  =  partial response, TRG 3  =  poor or no response. The representative pathology image of each grade was shown. (a) TRG 0, (b) TRG 1, (c) TRG 2, (d) TRG 3. Scale bar = 100 µm. I) A schematic drawing showing the effect of IFN‐γ‐mediated tryptophan consumption on the generation of W>F peptides in EBV‐positive GC. The effect was enhanced following hyperactivation of oncogenic mTOR/eIF4E pathway. The presence of W>F substitutions allows for their recognition and presentation on the cell surface, subsequently triggering T cell activation and eventual cell death. Data are presented as the mean ± SD. *P < 0.05, **P < 0.01.

Since the substitutants‐proteins are translational errors and therefore can be processed and presented by HLA‐I to the cell surface and caused T‐cell activation,^[^ ^12^ ^]^ we subsequently evaluated whether the IFN‐γ‐mediated production of W>F substitutants could influence the landscape of immunopeptides presented on the cell surface using a model peptide SIINFEKL from ovalbumin in GC cells. This system is based on the high affinity of SIINFEKL to class I molecules (mouse H‐2Kb), and the specific antibodies, 25D1.16, were used to detect class I/peptide complexes on the cell surface using flow cytometry analysis (**Figure** **7**A). Since SIINFEKL peptides contain phenylalanine (F) residues in the middle, we constructed two substituting mutants, SIINwEKL and SIINaEKL, in which F was replaced by tryptophane (w) or alanine (a), respectively. The H‐2Kb‐binding affinity of SIINFEKL and its mutants was predicted using the NetMHC algorithm (https://services.healthtech.dtu.dk/services/NetMHC‐4.0/) and the result indicated that SIINFEKL but not SIINwEKL or SIINaEKL bound to H‐2Kb with high affinity (Table S10, Supporting Information, affinity = 19.37 nM). Thus, the effect of W>F substitution on peptide presentation on the GC cell surface was assessed using flow cytometry. We established a stable H‐2Kb with SIINFEKL, SIINaEKL, or SIINwEKL overexpression in HGC27 and MKN45 cells using lentivirus vectors (Figure 7B). The overexpression efficiency was confirmed by western blot analysis with V5‐tag, tGFP, and Flag‐tag antibodies, which had a similar protein level of H‐2Kb and the engineered peptides (Figure 7C). The flow cytometry analysis revealed that cells expressing SIINFEKL peptides caused strong antibody recognition, but not SIINaEKL or SIINwEKL with the control condition. Moreover, IFN‐γ treatment upregulated the SIINFEKL signal in HGC27 and MKN45 cells with SIINwEKL overexpression, which was reversed by an IDO1 inhibitor. However, these alterations were not observed in SIINaEKL‐expressing cells (Figure 7D). Since EIF4E expression can contribute to the production of W>F substitutants, we further examined whether EIF4E affected the presentation of W>F peptides on the cell surface. HGC27 and MKN45 cells with SIINwEKL overexpression were transfected with scrambled or two EIF4E‐specific siRNAs and then cultured in a complete or ‐W medium. Flow cytometry analyses revealed that Trp depletion induced the upregulation of the SIINFEKL signal on the cell surface, while EIF4E silencing mitigated the signal intensity (Figure 7E).

In light of these findings, we aimed to explored the relationship between mTOR/eIF4E activation and immunotherapy response in an EBV‐positive GC cohort, which contains 16 patients received induction chemotherapy combined with anti‐PD‐1 immunotherapy before surgical resection. Immunohistochemistry analysis was conducted to examine the activation status of mTOR/eIF4E signaling by staining of eIF4E phosphorylation. Totally, 16 patients were categorized into the Low eIF4E phosphorylation group (*n* = 8) and High eIF4E phosphorylation group (*n* = 8) (Figure 7F; Table S11, Supporting Information). The immunotherapeutic response was evaluated based on the postoperative pathology according to NCCN guideline. The tumor regression grade (TRG 0–3) was defined according to the percentage of viable cancer cells in the resected primary tumor (Figure 7G).^[^ ^33^ ^]^ The High p‐eIF4E group had significantly higher major response rate (TRG0/1) compared to the Low p‐eIF4E group (Figure 7H), indicating that EBV‐positive GC patients with hyper‐phosphorylation level of eIF4E would gain better response from immunotherapy. Collectively, these data were consistent with the experimental results demonstrating that the Trp depletion‐mediated generation of W>F substitutants could be presented on the cell surface and caused T‐cell activation in gastric cancer.

To summarize, we observed a substantial abundance of a specific codon reassignment, W>F substitution, arising from Trp depletion in the EBV‐positive gastric cancer. The specific microenvironment with massive tumor‐infiltrating lymphocytes caused Trp consumption through IFN‐γ/IDO1 pathway. Further analysis of the difference of mRNA profiles between High‐ and Low‐W>F substitution group indicated that the activation of mTOR/eIF4E pathway boosted the production and presentation of abnormal W>F peptides. Intriguingly, although mTOR/eIF4E pathway was a pivotal driver for EBV‐positive gastric cancer, it also promoted the production of a substantial subset of abnormal W>F peptides, which exposed the fatal drawback of gastric cancer, increasing their susceptibility to the immune system. Therefore, these results suggested that EBV‐positive GC patients with hyperactivation of mTOR/eIF4E pathway were most likely to benefit from anti‐tumor immunotherapy (Figure 7I).

## 3 Discussion

The current study found that W>F substitutants were ubiquitous in EBV‐positive GC. In EBV‐positive GC, the extensive lymphocytic infiltration caused Trp deficiency and mainly led to codon reassignment translation, in which Trp was substituted by alanine, resulting in the production of numerous abnormal peptides. Since W>F substitutants belong to abnormal peptides, they can be presented on the cell surface and induce T cell immunity and could be strengthened by hyperactivation of the mTOR/eIF4E pathway. Therefore, we deem that EBV‐positive GC patients with mTOR/eIF4E hyperactivation were more likely to benefit from immunotherapy.

EBV‐positive GC is a unique type of GC, accounting for ≈10% of gastric cancer and consisting of cancer cells with monoclonal EB virus. It mainly presents with moderately‐ to poorly‐differentiated adenocarcinoma and harbors intense infiltration of lymphocytes.^[^ ^34^ ^]^ The EB virus has been demonstrated to induce oncogenesis in gastric epithelial cells by activating various cancer‐promoting signaling pathways, including Wnt, Akt/mTOR, cGAS/STING, and JAK2/STAT1 pathways.^[^ ^35^, ^36^, ^37^, ^38^ ^]^ We supposed that for EBV‐positive GC, EBV might activate the mTOR‐eIF4E pathway to different extents. Although the hyperactivation of mTOR‐eIF4E positively reinforced proliferation and metastasis in EBV‐positive GC, it also contributed to the generation of numerous W>F peptides and therefore exposed the fatal drawback of cancer cells, increasing their susceptibility to the immune system. On the other hand, heterogeneity still existed in the EBV‐positive GC and patients with the same TNM stage may have different clinical outcomes. Frontier research has been devoted to stratifying the patients with EBV‐positive GC into subgroups with different prognoses and treatment responses, so as to achieve individualized treatment.^[^ ^2^, ^39^ ^]^ Here, we also link the hyperactivation of the oncogenic mTOR/eIF4E pathway to the generation of abnormal peptides by codon reassignment, providing valuable insights into the development of novel biomarkers for identifying EBV‐positive patients sensitive to immunotherapy.

The initiation of ribosomal translation continues even under amino acid deficiency. Although IFN‐γ treatment or Trp depletion is decelerated, protein synthesis is not fully eliminated.^[^ ^11^ ^]^ The consistent loading of ribosomes on the mRNA and the unremitting ribosome elongation even in the absence of amino acid would increase the possibility of ribosome collision at the starving codons, thereby increasing the frequency of frameshifting and codon reassignment.^[^ ^40^, ^41^ ^]^ We observed that IFN‐γ treatment and Trp deficiency mainly induced codon reassignment (W>F substitution) in GC, while the frequency of ribosomal frameshifting events was rare. The mTOR/eIF4E is a key pathway that stimulates the assembly of eukaryotic translation initiation complexes and boosts protein translation. Our study demonstrated that the phosphorylation level of eIF4E could be a potential biomarker for immunotherapy response prediction of EBV‐positive GC patients. Further research determining the exact mechanism of the mTOR/eIF4E pathway in ribosomal substituting translation events will pave the way for understanding the role of the mTOR/eIF4E pathway in GC.

## 4 Conclusion

Although the W>F peptides produced by codon reassignment may only be a by‐product of the widespread imbalance in mRNA translation under amino acid starvation, they stimulated tumorigenicity by reducing the stress signals produced by ribosome collision.^[^ ^42^ ^]^ Thus, disorders in protein synthesis during cancer development may lead to enhanced ribosome collision events, resulting in stress signals to inhibit tumor progression. We speculate that the mTOR/eIF4E pathway could be activated to different extents after the infection of EB virus, and thus boost the generation of abnormal peptides. During the occurrence and progression of EBV‐positive GC, codon reassignment events seem to alleviate the cell cycle arrest and differentiation signals of stressed tumor cells. We further predict that in EBV‐positive GC, amino acid deprivation treatment might be used to enhance the sensitivity of immunotherapy by stimulating the synthesis of abnormal proteins.

## 5 Experimental Section

### Patients and Clinical Specimens

This study was approved by the Institutional Ethical Review Board of the Sun Yat‐sen University Cancer Center (GZR2023‐185), and written informed consent was obtained from all patients. All freshly frozen GC tissues were collected from Sun Yat‐sen University Cancer Center. All GC patients were confirmed histologically by two independent pathologists. The EBV status was detected by in situ hybridization (ISH). All patients had well‐documented clinical records and follow‐up information.

### Multiplex Immunofluorescence (m‐IF)

To evaluate the lymphocyte infiltration landscape in EBV‐positive GC, m‐IF staining was conducted as described previously.^[^ ^43^, ^44^ ^]^ In brief, formalin‐fixed paraffin‐embedded (FFPE) sections were incubated with an mIF labeling panel, which contains antibodies against CD20 (L26, Thermo Fisher Sci.), CD3 (D7A6E, Cell Signaling Technology), CD4 (EPR6855, Abcam), CD8 (C8/144B, Thermo Fisher Sci.), Pan‐Cytokeratin (Abcam), and their respective fluorophore. The 5‐color fluorescence channel and bright field channel were scanned by Cell DIVE Multiplexed Imaging System (Leica Microsystems, Germany), and DAPI was used as the location site and internal reference. The damage degree of tissue slices was controlled by HE images, and the background fluorescence images collected in each round were used for background correction. The quantitative analysis of the image was performed using the HALO software (Indica Labs, USA). The target information was extracted by accurate region of interest (ROI), and the expression pattern of each marker was recorded. The final results were expressed as cell densities from the total area analyzed (cell mm^−2^).

### Immunohistochemistry (IHC)

IHC was conducted as previously described.^[^ ^45^ ^]^ Briefly, paraffin‐embedded tissues were sliced into 5 mm segments. After deparaffinization, rehydration, and inactivation for endogenous peroxidase activity, the sections were subjected to antigen repair using 3% hydrogen peroxide and blocked with goat serum. The sections were incubated with anti‐IDO1 antibodies (1, 2000, Abcam) or anti‐eIF4E antibodies (Phosphor S209, 1, 200, Abcam) and then incubated with secondary antibodies. IHC staining was conducted using the Dako Envision System (Dako, Glostrup, Denmark) according to the manufacturer's instructions. The signal intensity representing the expression level was calculated using the Image Pro Plus software (version 6.0).

### RNA Extraction, Complementary DNA (cDNA) Synthesis, and Quantitative Real‐Time PCR (qRT‐PCR)

Total RNAs from clinical tissues or cell lines were extracted using TRIzol reagent (Invitrogen, CA, USA) following the manufacturer's protocol. Tissues or cells were washed with ice‐cold phosphate‐buffered saline (PBS) buffer and lysed in 1 ml TRIzol reagent. Approximately 200 µl of chloroform was added to each sample and mixed thoroughly. After centrifugation, the upper aqueous phase was collected, added to isopropanol, and then centrifuged. The precipitates were washed twice using 75% ethanol, air‐dried, and then diluted in ultrapure water. About 1000 ng of the extracted RNA was used for cDNA synthesis using a HiScriptIII one‐step Strand cDNA Synthesis Kit (Vazyme, Jiangsu, China) and supplied random hexamer primers. All qRT‐PCR reactions were conducted in triplicate using the SYBR Green Master Mix (Invitrogen) and the primers used were listed in Table S12 (Supporting Information). The housekeeping gene glyceraldehyde‐phosphate‐dehydrogenase (GAPDH) was used for normalization and the relative expression levels were calculated using the Comparative Ct method (ΔΔCt method).

### Cell Culture, Reagent, and Small Interfering RNA (siRNA) Transfection

The human gastric cell lines HGC27 and MKN45 and the immortalized human epithelial cell line GES1 were obtained from the American Type Culture Collection (ATCC). All cell lines were cultured in a Roswell Park Memorial Institute (RPMI)−1640 medium (Gibco, NY, USA) supplemented with 10% fetal bovine serum (FBS, Gibco). The cell lines were maintained in a humidified atmosphere at 37 °C with 5% CO_2_. The tryptophan‐free culture medium was purchased from Boster Biological Technology (Wuhan, China). IFN‐γ (Beyotime, Shanghai, China) was used at 250 U ml^−1^ for 48 h.

For siRNA‐mediated RNA interference, commercial siRNA sequences were designed and synthesized by RiboBio Company (RiboBio Co., Ltd., Guangzhou, China) (Table S13, Supporting Information). Transient siRNA transfection was performed using Lipofectamine RNAiMax (Invitrogen). HGC27 or MKN45 cells were seeded in six‐well plates one day before transfection and then transfected with specific siRNAs at a 50 nM final concentration. Transfection media were changed to normal complete culture media after 6 h of transfection. Cells were harvested 48–72 h post‐transfection and silencing was confirmed using qRT‐PCR and Western blot analysis.

### Western Blotting

Western blotting was performed as previously described.^[^ ^46^ ^]^ Briefly, cells were lysed with radioimmunoprecipitation assay (RIPA) lysis buffer (Beyotime, Shanghai, China) containing protease inhibitors (Roche, CA, USA). The protein concentration was determined by bicinchoninic acid assay (BCA) using the Pierce BCA Protein Assay Kit (Thermo Scientific, MA, USA) and ran on the 4–20% gradient polyacrylamide gels (20 mg per lane). The proteins were transferred to polyvinylidene fluoride (PVDF) membranes (Merck Millipore, Darmstadt, Germany), blocked with 5% nonfat milk, and then incubated at 4°C overnight with primary antibodies of IDO1 (ab211017, 1, 1000, Abcam), Total mTOR (T‐mTOR, 1, 5000, ab134903, Abcam), phosphor‐mTOR (p‐mTOR, S2448, ab109268, Abcam), Total 4EBP1 (T‐4EBP1, #9452, 1, 1000, CST), phosphor‐4EBP1 (p‐4EBP1, Thr37/46, #2855, 1, 1000, CST), Total EIF4E (T‐EIF4E, ab33766, 1, 1000, Abcam), phosphor‐EIF4E (p‐eIF4E, S209, ab76256, Abcam) or loading control α‐Tubulin (ab7291, 1, 5000, Abcam).), respectively. After washing off the non‐specific binders, blots were incubated with horseradish peroxidase (HRP)‐labeled corresponding secondary antibodies and then visualized using the enhanced chemiluminescence system, and blots were developed using the Pierce ECL Western Blotting Substrate Kit (Thermo Fisher).

### Detection of Trp and Kyn Concentration

The concentration of Trp and Kyn in GC tissues or cell lines was detected using high‐performance liquid chromatography with tandem mass spectrometry (LC‐MS/MS) of a multiple reaction monitoring (MRM) system (WininnovateoBio, Guangdong, China). Briefly, samples were lysed in 50 µL lysis buffer, followed by the addition of sulfosalicylic acid and 0.1% formic acid in water. After sonication and centrifugation, the supernatant was obtained and analyzed by LC‐MS/MS in positive ion mode. Amino acids were identified by comparing the retention times and MRM signature ion fragments of commercial standards. The quantification of Trp and Kyn concentration was performed using an external standard method. All samples were diluted to fall within the linear range of the standard concentration curve of the assay.

### Plasmid Construction and Lentiviral Infection

pLenti‐ATF4 and pTurboGFP‐B (Addgene) were used as templates to construct plasmids harboring ATF4^1‐64^ and turbo green fluorescent protein (tGFP) coding sequences, respectively. V5‐ ATF4^1‐64^‐tGFP (WT) and V5‐ ATF4^1‐64^‐+1 tGFP (+1 MUT) were generated by PCR and inserted in the pCDH‐Blast backbone as previously described.^[^ ^11^, ^12^ ^]^ A point mutation was introduced into the tGFP sequence from the V5‐ ATF4^1‐64^‐tGFP (WT) vector using the QuickMutation Site‐Directed Mutagenesis Kit (Beyotime) to generate V5‐ ATF4^1‐64^‐tGFP‐F26W (W>F MUT). The DNA sequence coding for the amino acid sequence LEQLESIINFEKL was cloned directly into the downstream of the tGFP sequence in the V5‐ATF4^1‐64^‐tGFP (WT) construct by PCR. SIINFEKL and ATF4^1‐64^‐point mutations were also generated by site‐directed mutagenesis and confirmed by sequencing. The H‐2Kb sequence was amplified from cDNA and cloned into the pCDH‐Hygro backbone. The sequences of all PCR primers were listed in Table S14 (Supporting Information).

For lentiviral infection, the lentivirus was produced using the HEK293T cells and third‐generation lentivirus vectors. Briefly, HEK293T cells were transfected with pCMV‐VSV‐G, pMDLg‐RRE, pRSV‐REV, and the target expression vectors using polyethyleneimine (PEI) in a 3, 1 ratio with total DNA. The supernatants were harvested 48 h post‐transfection, clarified by sterile 0.45‐µm filters, and concentrated by ultracentrifugation. Cells were then pretreated with polybrene (10 µg ml^−1^) for 20 min and infected by lentivirus for 6 h, and target cells were selected by antibiotic screening.

### Flow Cytometry

Flow cytometry analysis was performed on a CytoFLEX flow cytometer (Beckman Coulter). GC cells were seeded in six‐well plates and treated as necessary. Cells were washed twice with PBS, harvested by PBS‐EDTA treatment, and then washed with PBS containing 0.5% bovine serum albumin (BSA). For the detection of tGFP signals, cells were resuspended in PBS‐0.1% BSA for flow cytometry analysis. For the detection of SIINFEKL‐bound H‐2Kb peptides, cells were resuspended in PBS‐0.1% BSA with allophycocyanin‐conjugated 25D1.16 antibodies (1, 200, Biolegend, CA, USA) and then incubated on ice for 30 min in the dark. After washing out the unbound antibodies, cells were resuspended in PBS‐0.1% BSA for flow cytometry analysis.

### Generation and Analysis of Proteomics Data

Protein samples were prepared as described in previous studies.^[^ ^12^, ^47^ ^]^ Briefly, on the first day, MKN45 cells were seeded in 15 cm culture plates at 60% confluence. On the second day, they were subjected to treatment with either IFN‐γ or tryptophan‐free media. The cells that received DMSO or were cultured in complete media were used for mock treatment. 48 h after treatment, cells were incubated with MG132 (10 µM) for 4 h at 37°C to inhibit proteasomal degradation. Next, the cells were washed twice with ice‐cold PBS, harvested by scraping, and snap‐frozen using liquid nitrogen. Then, cells were then homogenously lysed in boiled guanidium lysis buffer, and then digested twice with trypsin. After desalting, the samples were completely dried and stored at −80°C until the peptide was fractionated. The preparation of clinical gastric cancer tissues and PDX samples was processed in a similar manner. Tissues were frozen through liquid nitrogen and ground using an electric homogenizer.

The samples were analyzed using a 2D liquid chromatograph mass spectrometer (LC‐MS)/MS. All proteins were identified using Maxquant software, with both PSM false positive (PSM FDR) and protein false positive (Protein FDR) rates being less than 0.01. To generate the substitutants database, human proteome was obtained from UNIPROT (https://www.uniprot.org/). The original mass spectrometry data (Raw data) were converted to MGF (Mascot Generic Format) format using the format conversion software MSConvert (v3.0.21307). The proteome search and identification software Xerotandem (v2015.04.01) was utilized to process and analyze the MGF format data. Xerotandem was configured with the following primary parameters, trypsin/P digestion, with tryptophan replaced by phenylalanine as a variable modification resulting in a mass shift of −39.010899 on the affected amino acid. Oxidation (M) was also designated as a variable modification, while Carbamidomethyl (C) was set as a fixed modification. The first‐order mass tolerance was 10 ppm, and the second‐order mass tolerance was 0.02 Da. The UNIPROT human protein sequence library was used as the reference database. Notably, the results from Xerotandem were further refined and enhanced for accuracy and sensitivity through subsequent processing with Percolator.^[^ ^42^, ^43^, ^44^ ^]^

### Bioinformatics Analysis

Gene set enrichment analysis (GSEA) was conducted on the gene expression profile from the GSE122401 dataset to determine the genes and pathways related to the generation of W>F substitutants, utilizing the GSEA software with MSigDB v6.2 (https://www.broadinstitute.org/gsea). The genes were ranked based on the 1000 permutations of the gene sets and log2 ratio of classes. The normalized enrichment score (NES) for each gene set was considered as the main GSEA statistic results. The calculated P‐values was subjected to FDR correction, with a threshold of FDR < 0.05 applied to select significant items. The variance in the molecular characterization of tumor‐immune interactions between the high and low W>F substituent groups was assessed using the Tumor Immune Estimation Resource (TIMER, https://cistrome.shinyapps.io/timer/).

### Patient‐Derived Xenograft (PDX)

For establishing the PDX, patient tumor explants were obtained from surgical resections of EBV‐positive GC patients at the Sun Yat‐sen University Cancer Center (SYSUCC, China). The written informed consent was obtained from all patients before sample collection. Briefly, patient‐derived tumor samples were cut up separately into fine pieces, and loaded into 1 ml syringes with 14‐gauge needles. The homogenized tumor tissues were inoculated subcutaneously into the right flanks of NCG mice while the animals were under anesthesia. Once tumor volume reached 1500 mm^3^, the mice were sacrificed. Then, the tumors were collected and a portion of the tumor was engrafted in new mice. The primary tumors were implanted into the first generation of mice, which were defined as F1 mice. For the successful engraftment cases, the tumors (F1) were removed for serially implanted into second‐ (F2) and third‐generation (F3) mice.

### Cell Proliferation Analysis

Cell proliferation ability was measured using a Cell Counting Kit‐8 (CCK‐8, Dojindo, Tokyo, Japan) assay according to the manufacturer's instructions. The cells were subjected to siRNAs transfection, and 2000 GC cells were seeded per well in a 96‐well plate on the first day (Day 0). The CCK‐8 working solution was prepared using the culture medium and CCK‐8 reagent (v/v = 10, 1). From day 1 to day 5, the culture medium was refreshed with CCK‐8 working solution and incubated for 4 h in a humidified incubator at 37 °C. A microplate reader was used to read the absorbance at wavelength of 450 nm and the results were used to construct cell proliferation curves.

### Transwell Migration and Invasion Assay

Transwell assay was performed to evaluate the cell migration and invasion ability using the 24‐well Transwell inserts (Corning, NY, USA). 72 h after transfection, HGC27 and MKN45 cells were resuspended in 200 µl serum‐free RPMI‐1640 medium with (invasion assay) or without (migration assay) Matrigel (Corning), and added to the upper chamber of the Transwell insert. The lower chamber was loaded with 600 µl of culture medium supplemented with 10% FBS. Following 24 or 48 h of incubation, cells that migrated or invaded to the underside of the Transwell membrane were immobilized with formaldehyde and then stained with hematoxylin. The images were captured using an Inverted microscope (Olympus) and cells were counted at 200× magnification.

### Statistical Analyses

Statistical analyses and data presentation were performed using GraphPad Prism (version 8.0, GraphPad Inc., CA, USA), SPSS (version 23.0, IBM Corp., NY, USA), and R (version 3.6) software. Data were presented as the mean ± standard deviation (SD). Data between the two groups were conducted using the paired or unpaired Student's *t*‐test or the *chi*‐squared test. For multiple groups, One‐way analysis of variance (ANOVA) was performed followed by the Bonferroni test. Pearson correlation tool was employed to perform correlation analyses. All experiments were run as triplicates and three times independently. A *P*‐value of less than 0.05 was considered statistically significant.

### Ethics Approval

This study was approved by the Institutional Review Board of Sun Yat‐Sen University Cancer Center (GZR2023‐185). All animal experiments were approved by the Institutional Animal Care and Use Committee, Sun Yat‐sen University Cancer Center (L102012023000K).

## Conflict of Interest

The authors declare no conflict of interest.

## Author Contributions

Z.‐Q.Z., C.‐R.Z., C.‐Z.W., and X.‐J.C. contributed equally to this work. Z.Q.Z., and Y.L.L. performed conceptualization. Z.Q.Z., C.R.Z., C.Z.W., X.J.C., G.M.C., R.C.N., Z.W.C., F.Y.Z., and Y.F.L. performed data production, analysis and investigation. Z.Q.Z., C.R.Z., C.Z.W., Z.W.Z., Y.M.C., and Y.L.L. wrote, review and edited the original draft. T.L.Y., Y.M.C., and Z.W.Z. performed supervision. Z.Q.Z., T.L.Y., Y.M.C., and Z.W.Z. performed funding acquisition. The authors read and approved the final manuscript.

## Supporting information

Supporting Information

Supplementary Table S1

## Data Availability

The data that support the findings of this study are available in the supplementary material of this article.
