# Supplementary material for: Hyperactivation of mTOR/eIF4E Signaling Pathway Promotes the Production of Tryptophan‐To‐Phenylalanine Substitutants in EBV‐Positive Gastric Cancer
Source: Adv Sci (Weinh). 2024 Jul 12;11(35):2402284. doi: 10.1002/advs.202402284 (PMC11425274; doi:10.1002/advs.202402284)
Supplement: Supplementary file 1 — Supporting Information [file ADVS-11-2402284-s001.pdf]

Supplementary Figure S1

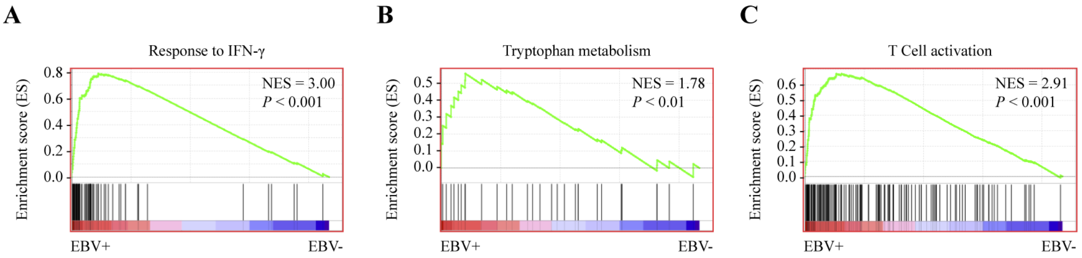

Supplementary Figure S1. IFN-γ-mediated Trp consumption in EBV-positive gastric cancer.

A–C. GSEA based on the transcriptome profile from GEO dataset (GSE122402) which contains 74 cases of EBV-negative GC tissues and 6 cases of EBV-positive GC tissues revealed the enrichment of IFN-γ response (**A**), Trp metabolism (**B**), and T cell activation (**C**) pathways in EBV-positive GC tissues.

Supplementary Figure S2

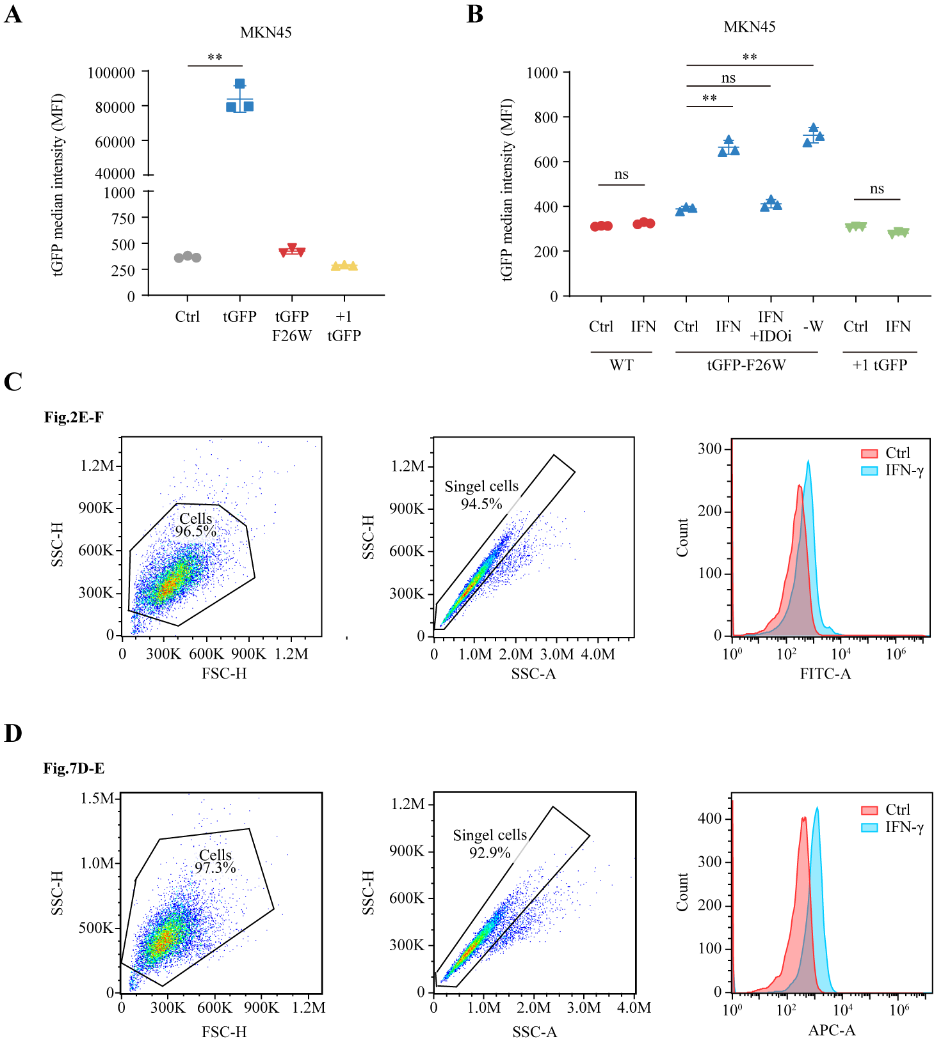

Supplementary Figure S2. Tryptophan depletion caused tryptophan-to-phenylalanine substitution in GC cell lines

A. The tGFP signals were measured by flow cytometry analysis in MKN45 cells stably expressing empty vector, tGFP, tGFP-F26W, or +1 tGFP reporters, respectively. **B.** MKN45 cells stably expressing the empty vector, tGFP-F26W, or +1 tGFP reporters were cultured and treated as indicated in the figures, and subjected for flow cytometry analysis to detect the tGFP signaling intensity. **C.** Gating strategy for flow cytometry analysis in Figure 2E-F. **D.** Gating strategy for flow cytometry analysis in Figure 7D-E. Data are presented as the mean ± SD. **$P < 0.01$, ns = not significant.

Supplementary Figure S3

A

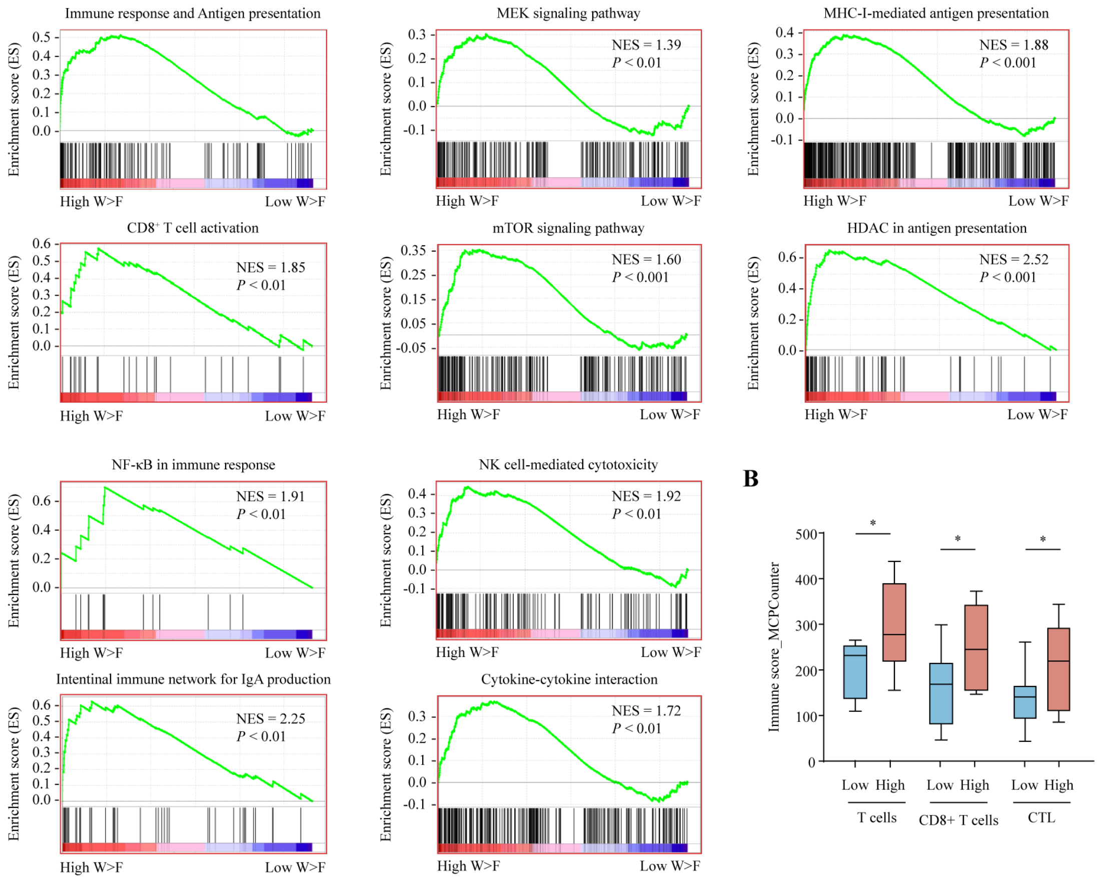

Supplementary Figure S3. W>F substitutions was associated with T cell activation in EBV-positive GC.

A. Samples from the CPTAC-STAD dataset were classified into high- or low-W>F according to the number of W>F substitution events. GSEA was performed to explore the potential biological functions in the high- or low-W>F groups in STAD tissues. **B.** The landscape of immune cell infiltration in high- or low-W>F groups was evaluated by TIMER platform. *$P < 0.05$

Supplementary Figure S4

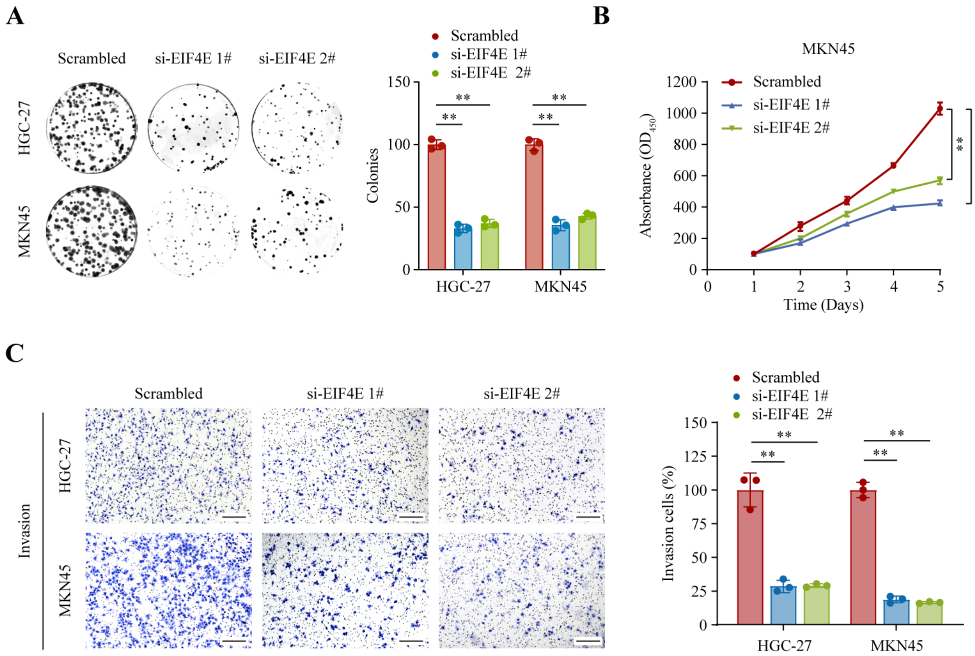

Supplementary Figure S4. Oncogenic mTOR/eIF4E pathway boosts the generation of W>F substitutants in GC cells.

A-B. Colony formation assay (**A**) and CCK-8 analysis (**B**) were performed to assess the proliferation ability of MKN45 cells transfected with control siRNA or EIF4E siRNAs.

C. After knockdown of EIF4E with two individual siRNAs, the migration and invasion ability of MKN45 cells was detected by Transwell assay. The representative images (left panel) and the counts migratory/invasive cells (right panel) in the Transwell chambers were shown. Data are presented as the mean ± SD. **$P < 0.01$.

Supplementary Figure S5

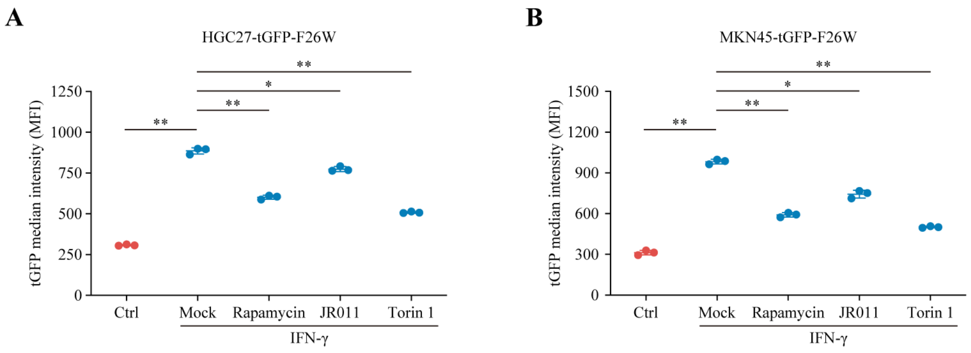

Supplementary Figure S5. Inhibiting of mTOR pathway alleviated the generation of W>F substitutants.

A–B. HGC27 and MKN45 cells with tGFP-F26W stable expression were cultured in medium with Ctrl or IFN-γ, and treated with Rapamycin (10 nM), JR-AB2-011 (10 μM), or Torin 1 (200 nM). Then, MG132 (10 μM) was added to inhibit proteasome and cells were sent for clow cytometry analysis to measure the signal intensity of tGFP in HGC27 (**A**) and MKN45 (**B**) cells. Data are presented as the mean ± SD. *$P < 0.05$, **$P < 0.01$.
